# Pneumococcal meningitis outbreak and associated factors in six districts of Brong Ahafo region, Ghana, 2016

**DOI:** 10.1186/s12889-018-5529-z

**Published:** 2018-06-22

**Authors:** Timothy Letsa, Charles Lwanga Noora, George Khumalo Kuma, Ernest Asiedu, Gideon Kye-Duodu, Edwin Afari, Osei Afreh Kuffour, Joseph Opare, Kofi Mensah Nyarko, Donne Kofi Ameme, Emmanuel G. Bachan, Kofi Issah, Franklin Aseidu-Bekoe, Moses Aikins, Ernest Kenu

**Affiliations:** 1 School of Public Health, University of Ghana, Accra, Ghana; 2 Ghana Field Epidemiology and Laboratory Training Program, Accra, Ghana; 3 Ghana Health Service, Sunyani Municipal Hospital, PMB, Sunyani, Ghana

**Keywords:** Pneumococcal meningitis, Tain district, Brong Ahafo region, Ghana

## Abstract

**Background:**

Meningitis, a disease of the Central Nervous System is described as inflammation of the covering of the brain and spinal cord (meninges). It is characterised by fever, severe headache, nausea, vomiting, stiff neck, photophobia, altered consciousness, convulsion/seizures and coma. In December, 2015, twelve suspected cases of meningitis were reported in Tain district in Brong Ahafo region (BAR). Subsequently, dozens of suspected cases were hospitalized in five district hospitals in BAR. We investigated to determine the magnitude, causative agent and risk factors for the disease transmission.

**Methods:**

A community-based 1:2 case-control study (with 126 individuals) was conducted form 10/12/15 to 26/4/16 in 27 districts of Brong-Ahafo Region, Ghana. We defined suspected meningitis cases as people presenting with sudden headache and fevers (Temp> 38.0 °C) in combination with one of the following signs: neck stiffness, altered consciousness, convulsions, bulging fontanelle (infants) and other meningeal signs. Controls were selected from the same neighbourhood and defined as individuals with no overt meningitis signs/symptoms. We collected CSF samples and performed serological testing using Pastorex-Meningitis-Kit and culture for bacterial isolation. Moreover, structured questionnaires were used to collect data on socio-demographics, living conditions, health status and other risk factors. We conducted univariate data analysis and logistic regressions to study disease-exposure associations using Stata 15.

**Results:**

A total of 969 suspected cases with 85 deaths (CFR = 9.0%) were recorded between December, 2015 and March, 2016. Majority, 55.9% (542/969) were females aged between 10 months-74 years (median 20 years, IQR; 14-34). Of the 969 cases, 141 were confirmed by Laboratory test with *Streptococcus pneumoniae* identified as the causative agent. Cases were reported in 20 districts but 6 of these districts reported cases above threshold levels. The outbreak peaked in week 6 with 178 cases. Overall attack rate (AR) was 235.0/100,000 population. District specific ARs were; Tain; 143.6/100,000, Wenchi; 110.0/100,000, Techiman; 46.6/100,000, Jaman North; 382.3/100,000 and Nkoranza South; 86.4/100,000. Female and male specific ARs were 251.3/100,000 and 214.5/100,000 respectively. Age group 10-19 years were most affected 33.8% (317/940). We identified sore throat [aOR = 5.2, 95% (CI 1.1-26.1)] and alcohol use [aOR = 9.1, 95%(CI 1.4-55.7)] as factors associated with the disease transmission.

**Conclusion:**

Meningitis outbreak due to *Streptococcus pneumoniae* was established in BAR. Upper respiratory tract infection and alcohol use were associated with the outbreak. Mass campaigns on healthy living habits, signs and symptoms of meningitis as well as the need for early reporting were some of the control measures instituted. Moreover, we recommend Pneumococcal vaccination in BAR to prevent future outbreaks.

**Electronic supplementary material:**

The online version of this article (10.1186/s12889-018-5529-z) contains supplementary material, which is available to authorized users.

## Background

Meningitis is a disease of the Central Nervous System (CNS) and described as inflammation of the covering of the brain and spinal cord (meninges) [1]. The disease has several causes including virus and bacteria and is characterised by fever, severe headache, nausea, vomiting, stiff neck, photophobia, altered consciousness, convulsion/seizures and coma [1]. Without treatment, the case-fatality rate can be as high as 70%, while 10-20% of survivors may be left with permanent sequelae including hearing loss, mental retardation and non-functional limbs [2, 3]. *Streptococcus pneumoniae*, *Neisseria meningitides and Haemophilus influenza accounts for 80% of* all causes of bacterial meningitis [3]. Incubation periods for *S. pneumoniae* is between 2 and 4 days. The spread from person to person is by close contact with respiratory secretions [1, 4, 5]. The leading cause of acute bacterial meningitis in adults is *S. pneumoniae* with an average mortality rate of 25% despite effective antibiotic therapy and modern intensive care facilities [6].

Annually, more than 1.2 million cases of bacterial meningitis are estimated to occur worldwide but incidence and case-fatality rates for the disease vary by region, country, pathogen, and age group [2]. WHO estimates that, annual global incidence of pneumococcal meningitis is between 1 and 2 cases per 100,000 populations [2]. Global incidence of meningitis is highest in a region of Sub-Saharan African known as the “meningitis belt” which extends from Senegal to Ethiopia, and is characterized by seasonal epidemics during the dry season [5]. Ghana lies within the African meningitis belt which accounts for the highest burden of meningitis worldwide [7]. The Northern, Upper East, Upper West, and the Northern parts of Brong Ahafo and Volta Regions in Ghana lie within this belt. Ghana and other countries of this region, have occasionally experienced large scale epidemics caused by *N. meningitides* sero-groups A, C and more recently W [7], however there has been limited outbreaks due to *S. pneumoniae* in countries within the meningitis belt. This probably accounts for the few focal studies in pneumococcal meningitis. Environmental factors including climate change and rainfall patterns, low absolute humidity and dust are known risk factors for the spread of bacterial meningitis [8, 9]. According to WHO, travel and migration are known to facilitate the spread of bacteria that cause meningitis [3]. Over 3000 meningitis cases and 400 deaths were reported in Ghana between 2010 and 2015 [10]. More than 95% of Ghana’ meningitis burden is due to Neisseria meningitides (Nm). However, there have been few reported outbreaks of Streptococcus meningitis in recent times in Ghana [10].

On 31st December, 2015 the District Director of health services in Tain was alerted of a suspected meningitis outbreak in the Tain district. Three residents from a small farming community, Brohani in the Tain district of the Brong Ahafo region of Ghana presented to the Brohani health centre with complaints of fever, headache and neck pain and they were referred to the district hospital. Two of them died shortly upon arrival at the hospital. Subsequently, dozens of people across neighbouring districts presented at other health facilities with similar symptoms. The Regional Public Health unit upon reports from affected District Health Directorates, suspected an outbreak of meningitis. A team of residents and alumni of the Ghana Field Epidemiology and Laboratory Training Programme (GFELTP) was constituted to investigate the suspected outbreak. We report the cause, magnitude and factors associated with the spread of the disease to inform appropriate control and preventive measures.

## Methods

### Outbreak setting

**Fig. 1:**
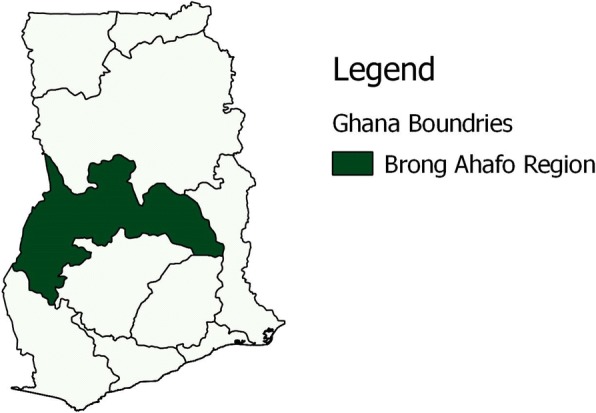
Map of Brong Ahafo Region, Ghana, Field work, 2016

The outbreak occurred in six out of 27 districts in the Brong Ahafo Region (BAR) of Ghana (Fig. 1). The region with a territorial size of 39,557 km^2^ is the second largest in Ghana and located in the central part of the country with a projected population of 2.6 million [11]. The Region is further divided into 27 administrative districts [11]. There are two main ecological zones in the region; the forest and savanna transition zones which makes it vulnerable for the spread of disease of epidemic potential from the northern or southern parts of the country. The BAR much like all regions in Ghana runs an integrated disease surveillance system as a strategy to detect and respond effectively to diseases of public health importance, or epidemic potential. There is one regional hospital, 22 District hospitals, 131 health centres, and 467 CHPS (Community Based Health Planning and Services) facilities conducting facility based disease surveillance. The facility based disease surveillance system in the region is complemented by a community based surveillance system consisting of 4000 volunteers in communities in the region tasked to report unusual health events on a monthly basis to the Sub-district Health Management Teams [12].

### Study design

We conducted a descriptive study to identify factors associated with the spread of pneumococcal meningitis in BAR between December 2015 and 6th April, 2016. Participants included all persons living in Brong Ahafo Region who had been confirmed as having meningitis or has an epidemiological linkage with a confirmed case of meninigitis and were line listed (collection of data in rows and columns with variables) in the database of the Brong Ahafo Regional Public Health Unit of meningitis.

### Sample size and sampling procedure

We conducted a 1: 2 unmatched case control study in affected communities using the entire region as the study population. Using the Statcalc’ utility feature of epi-info statistical software and based on sample size formula for comparing proportions, we calculated a minimum sample size at confidence level of 95%, power of 80%, expected exposure (upper respiratory tract infection) frequency in controls of 24%, expected exposure frequency in cases (upper respiratory tract infection) of 49.7%, case to control ratio of 1:2 and an odds ratio of 3.1 (for a risk factor on which intervention would have a significant impact) to achieve 42 cases and 84 controls.

### Selection of cases and contols

Cases were selected from health facilities within the region. We obtained daily line lists of cases who were mostly on admission and interviewed until we met our required sample size. A case of meningitis was defined as *“any person living in the BAR, reporting with sudden onset of fever and headache and one of the following signs: neck stiffness, altered consciousness, convulsions, bulging fontanelle (infants) and other meningeal signs from the 9*^*th*^ *December, 2015”* with laboratory confirmation (positive for bacteria-causing meningitis).

Controls were defined as persons living in the same community with cases but who did not show symptoms of meningitis during the same period. Cases were located based on the addresses provided on the line list and in folders of cases at the hospitals.

Two neigborhood controls were randomly selected for each case interviewed. Based on the house address of cases interviewed, we followed up to communities where cases came from to select controls. In the absence of a proper sampling frame (street addresses, post code etc), the controls were selected for each case by a member of the investigative team standing in front of the house case and spinning a pen to determine a starting direction. One control was selected from one house on either sides of the pen for each control and interviewed. The study was explained to the household by the field workers and if they agreed to participate, workers selected an available household member and interviewed the individual as a control for the study. Individuals were excluded from being controls if they reported symptoms of meningitis from 9th December, 2015 (two incubation periods) to 6th January, 2016.

A standardized questionnaire (Additional file 1) was administered to cases and controls in their native tongue by members of the investigative team and trained field technicians recruited from the six district Health Directorates. The questionnaire collected basic demographic information, travel history and other risk factors for meningitis, from 9th December, 2015 to 6th January, 2016. In cases where a child was the study subject, questions were asked to an adult within the household (typically a family member) who had knowledge of the child’s activities after obtaining an informed consent and assent (in the case of children). Geographical coordinates of landmarks and residences of cases were taken with a GPS receiver to construct spot maps. We surveyed the communities in each district to assess ventilation of houses, hygiene practices and sanitation conditions in the communities. We also interviewed controls to determine number of people living in a room. We interacted with the regional and district Meteorological officers.

### Data collection methods

We interacted and interviewed Municipal Coordinating Directors, Municipal Directors of Health Services, Disease Control Officers, Health Information Officer and other key health officials of all Hospitals and health centres in Brong Ahafo. The investigation team obtained and reviewed available line lists. We trained Disease Surveillance Officers on data collection methods and the use of Geographical Positioning System (GPS) receivers to pick coordinates of residences of case patients and controls, health facilities and other landmarks and also conducted health education on the strict use of the case definition by health workers and sensitized them on active case finding. We conducted active case search and reviewed medical records at six district hospitals and 18 health centers. Data abstracted included age, sex, occupation, address, any travel history, date of onset of symptoms, date of presenting to the health facility, and date of outcome of admission.

### Data analysis

Data was entered into and analysed in Epi-info data version 3.5.4. We performed descriptive analysis of the outbreak data by person, place and time. Univariate analyses were expressed as frequency distributions, mean (±SD) and rates (as appropriate). We calculated the age and sex specific attack rates, incidences by age-group and sex, drew an epidemic curve and constructed a spot map representing the spread of the disease in the communites. Binary and multiple logistics regressions were done to assess factors associated with the meningitis outbreak and presented as odds ratios with their corresponding 95% confidence intervals.

### Thresholds determinations

We defined and determine alert (A level of incidence that triggers action to prepare for an epidemic, including strengthening surveillance, confirming cases, distributing treatment protocols and informing the authorities) and epidemic (A higher level of incidence that triggers an epidemic response, including mass vaccination, antibiotic distribution and raising public awareness) thesholds of meningities based on WHO [13] standard guidelines for meningities.

## Results

### Descriptive epidemiology

#### Magnitude and geographical spread

**Fig. 2:**
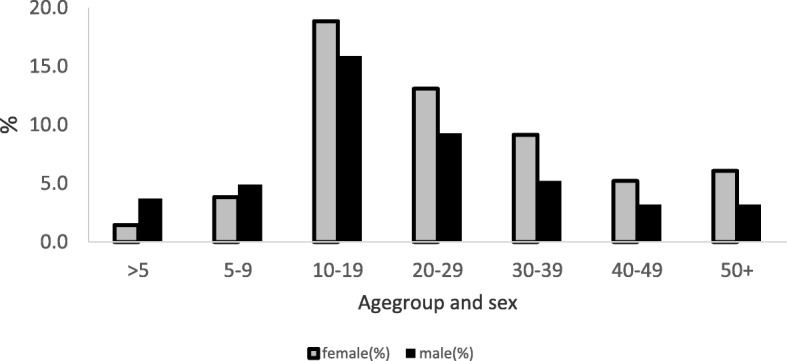
Age group and sex distribution of meningitis case, BAR, 2016

Nine hundred and seventy four meningitis cases were reported between December 2015 and April 2016. More than half, 55.9% (542/974) were females. The overall attack rate was 235/100,000 with 9.0 case fatality rate. Sex specific attack rates were 251.3/100,000 and 214.5/100,000 populations for females and males respectively. The median age of the case-patients was 20 years (IQR: 14-34 years). The most affected age group was 10-19 followed by 20-29 (Fig. 2). More females were affected in the most affected age groups (10-19, 20-19).

**Fig. 3:**
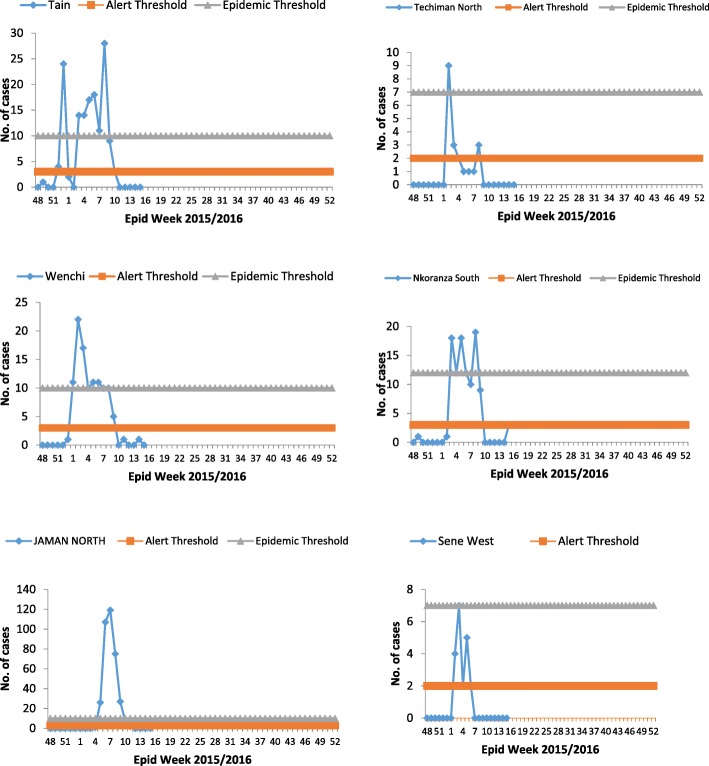
Alert and epidemic thresholds of meningitis by weeks in district reporting outbreaks, BAR

**Fig. 4:**
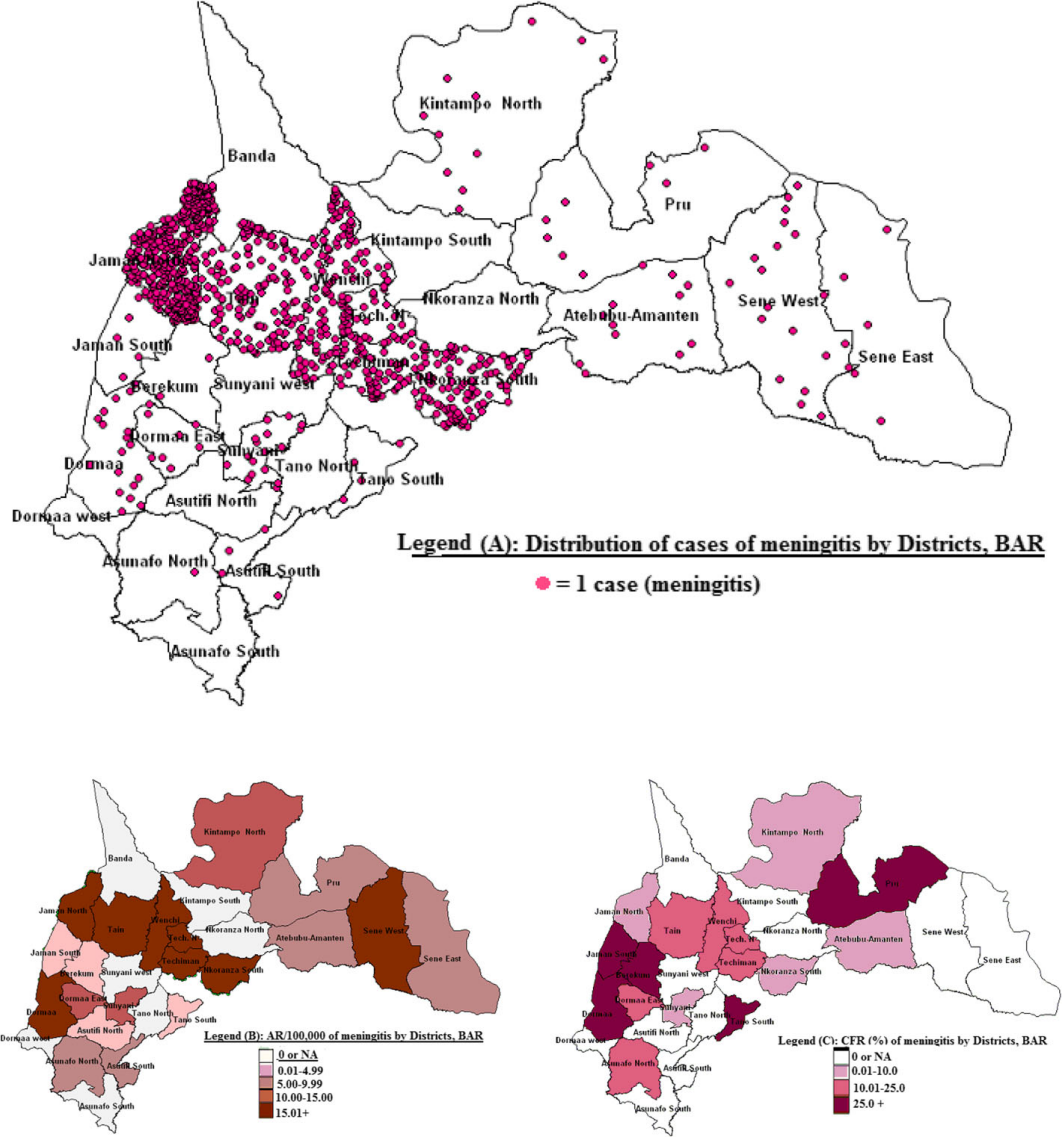
Cases (top), Attack Rate (AR) (lower left) and CFR (lower right) of meningitis, BAR 2016, Field work, 2016

Twenty out of 27 districts in the region all reported cases of meningitis. Cases were largely clustered in six districts (Fig. 3). They included; Jaman North 37.5% (364/974), Tain 14.9% (145/974), Wenchi 11.3% (110/974), Nkoranza 10.6% (103/974) and Techiman 8.1% (79/974). Attack rates were high in Jaman North 382.3, Tain 143.6, Wenchi 110, Nkoranza 86.4 and Techiman 46.6 per 100,000 populations (Fig. 4). Others include; Sene West 29.9/100,000, Techiman North 29.5/100,000, and Dormaa 21.0/100,000. Case fatality rates in Pru, Dormaa, Jaman South, Berekum and Tano South were above 25%.

#### Incidence of meningitis in BAR by time of onset

**Fig. 5:**
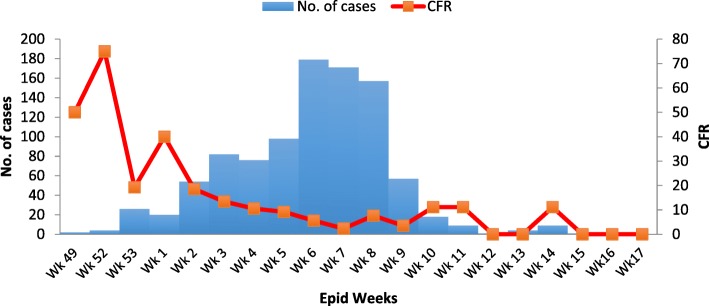
incidence of meningitis by time of onset, Brong Ahafo Region, December 2015-April, 2016

The index case was a 70 year old woman who hail from Brohani, she presented to the Brohani CHPS center on the 2nd of December, 2015 with fever, headache and neck pain 3 days after she had visited her cassava farm. She had not travelled nor received any visitor in the past 2 weeks. She was treated as a case of malaria and discharged. Her condition did not improve and she died a week later. Her grandson a 14 year old boy also died 10 days later (24th December, 2015). The epidemic curve of the outbreak shows a propagated source. Two cases occurred in epidemiological week 49 followed by four cases in week two. The number of cases rose sharply to a peak at epidemic week six with 171 cases and declined with the last case recorded in week 15 (Fig. 5).

#### Analytical epidemiology

Sore throat and alcohol use were identified as key risk factors in the current outbreak (Table 1). The odds of having sore throat prior to the outbreak and developing meningitis was increased by 5.2 times compared to individuals with no sore throat [aOR = 5.2, 95% CI (1.1-26.1)]. Similarly, alcohol users had an odds of 9.1 of developing meningitis compared with non-alcohol users, [aOR = 9.1, 95% CI (1.4-55.7)]. There were associations between age groups, occupation, history of travel and number of persons per room, however these were significant statistically (Table 1).

**Table 1:** Factors associated with the spread of pneumococcal meningitis, BAR, 2016

Factor	Cases (*n* = 40)	Controls (*n* = 80)	cOR (95% CI)	*P*-Value	aOR (95% CI)	
Sex						
Male	11(23.4)	36(73.6)	1.0		1.0	
Female	31(38.7)	49(61.3)	2.1(0.9-4.7))	0.08**	4.9(0.9-26.4)	0.06
Age group (yrs)						
≥ 19	16(69.6)	7(30.4)	1.0		1.0	
20-39	14(24.1)	44(75.9)	0.1(0.1-0.4)	< 0.001*	0.5(0.1-5.5)	0.57
≥ 40	12(26.1)	34(73.9)	0.2(0.1-0.5)	< 0.01*	0.8(0.1-12.1)	0.89
Occupation						
Student	17(56.7)	13(43.3)	1.0		1.0	
Farming	20(29.0)	49(71.0)	0.3 (0.2-0.8)	0.01*	0.3(0.1-3.4)	0.36
Other	5(17.9)	23(82.1)	0.2 (0.1-0.5)	< 0.01*	0.2(0.01-5.4)	0.36
Education						
None	16(33.3)	32(66.7)	1.0		1.0	
Primary	21(47.7)	23(52.3)	1.8(0.8-4.2)	1.00	2.1(0.4-10.6)	0.38
Other	3(9.7)	28(90.3)	0.2(0.1-0.9)	0.02*	0.1(0.01-1.5)	0.08
Religion						
Christian	36(32.2)	74(67.3)	1.0		1.0	
Muslim	5(31.3)	11(68.8)	0.9(0.3-2.9)	0.91	1.4(0.4-4.5)	0.55
Travel history						
No travel	15(26.8)	41(73.2)	1.0		1.0	
Travelled within last 3 weeks	26(37.7)	43(62.3)	1.7 (0.7 – 3.6)	0.19**	1.8(0.5-7.3)	0.38
Cough history						
No cough	16(29.6)	38(70.4)	1.0		1.0	
Had cough	7(41.2)	10(58.8)	1.7(0.5-5.1)	0.38	2.0(0.5-6.4)	0.33
Sore throat history						
No sore throat	17(26.1)	48(73.9}	1.0		1.0	
Had sore throat	10(62.5)	6(37.5)	4.7(1.3-15.9)	< 0.01*	5.2(1.1-26.1)	0.04*
Alcohol use						
No alcohol	23(27.4)	61(72.6)	1.0		1.0	
Takes alcohol	13(37.1)	22(62.9)	1.6(0.7-3.6)	0.24**	9.1(1.4-55.7)	0.02*
Windows per room						
0 – 1	15(27.8)	39(72.2)	1.0		1.0	
2 or more	23(33.8)	45(66.2)	0.8 (0.3 – 1.6)	0.47	0.4(0.1-1.8)	0.22
Persons per room						
1-2	9(23.9)	30(76.9)	1.0		1.0	
More than 2	33(37.9)	54(62.1)	2.0(0.8-4.9)	0.10**	0.3(0.1-1.80	0.19
Use firewood						
No firewood	6(31.6)	13(68.4)	1.0		1.0	
Use firewood	30(30.3)	69(69.7)	0.9(0.3-2.7)	0.91	1.2(0.4-3.2)	0.21

Note: * = *p* < 0.05, ** = *p* < 0.25

#### Environment findings

We found that, most houses in the urban communities had two windows whereas those in the rural communities had one or no window with overall mean of 1.8 SD ±1.3 windows per room. The mean number of persons living in a room was 3.1 SD ± 1.5. Major roads within the districts and towns were tarred, however most of the roads leading to communities within the sub-districts were untarred and dusty. According to the Regional meteorological officer, rainfall patterns with regards to the period for both the raining season and the dry season as we have in BAR has changed especially over than last five years. There are now shorter seasons (April to October) and fewer rains compare to ten years ago. The hamathan season has become longer and intense (from December to February) with hash dry weather and high temperatures sometimes above 40 °C.

#### Public health actions

Regional and District Health Management Teams collaborated with the political leaders (district assemblies) and initiated a prompt investigation into the outbreak. Lumber puncture was conducted for more than 80% (840) of all suspected cases. Chemoprophylaxis in the form of azithromycin were administered by disease control officers and field technicians to all primary and secondary contacts of confirmed cases of meningitis in the region. Health promotion officers conducted health education on causes, signs and symptoms, and prevention of meningitis in schools, churches, mosques, market places and on radio emphasizing the need for early reporting upon onset of symptoms.

## Discussion

In 2003, an enhanced meningitis surveillance network was established across the meningitis belt of sub-Saharan Africa to rapidly collect, disseminate, and use district weekly data on meningitis incidence in order to detect and manage outbreaks [10]. There have been outbreaks of meningitis within Ghana’s meningitis belt almost every year, more than 97% due to *N. meningitides*.

Findings from this study established an outbreak of meningitis due to *S. pneumoniae*. The meningitis outbreak started from a small farming village, Brohani in the Tain district of the BAR which eventually spread to other districts of the region. Cases of meningitis were reported in almost all districts of the region, however only six districts; Jaman North, Tain, Nkoranza South, Techiman and Wenchi recorded outbreaks. The meningitis outbreak of 2015/2016 with a total of 2184 (971 in Brong Ahafo Region) cases is the largest to be recorded in modern times in comparison to 1619 cases in the 1996/1997 which is on record as the largest in Ghana [10]. The propagated nature of the outbreak meant that there were a number of factors that acted favourably to enhance the rapid spread in a region which traditionally lies outside the meningitis belt of Africa , and might be considered to have large populations of susceptibles.

The period of the outbreak (December to March) is the normal meningitis season in Ghana and was characterised by very dusty and cold winds, and very low humidity [11] making it conductive for the development of upper respiratory tract infections, increased carrier rates of organisms [14] and leading to simultaneous outbreaks in the various districts. According to WHO [3], the conditions described above have been linked to *N meningitides*. Our study is limited in the accurate use of case definitions to detect patients with meningitis giving rise to a possible over-diagnosis or missing other cases of the disease. Secondly the sensitivity and specificity of the various laboratory tests did not undergo quality control using more efficacious tests thus giving the possibility of a number of false positive or false negative results.

With poor record keeping characterizing the documentation procedures of the Ghana Health Service and limited data cleaning conducted during this study we could not rule out missing some cases/double counting of cases and also misclassifying some deaths as being due to meningitis due to paucity of patient information gathered by the clinicians and other health staff. Despite the above limitations we have no doubt the data gathered and presented in this study offers readers and researchers an opportunity to have an insight into the nature and extent of the 2015/2016 meningitis outbreak in the Brong Ahafo Region of Ghana.

The high attack rates (234.8/100,000) as compared to rates between 10 and 120% in the traditional meningitis belt of Ghana [10, 13, 15] confirm the susceptibility and vulnerability of the population of the region. The high case fatality of more than 25% in some districts confirms work carried on by other researchers where the case fatality ranges between 17 and 30% [10].

The high level of susceptibility combined with a failure in surveillance in the Jaman north district might account for the highest attack rate of 382.3/100,000 and recording 37.5% of the 971 cases in the outbreak***.*** On the other hand the Brohani community and the Tain district from where the index case was recorded were successful in preventing further spread probably due to improved case management and enhanced surveillance and ending up with only 14.9% of cases but an attack rate of 143.6/100,000.

The epi curve showed that cases were recorded for 18 epidemiological weeks (week 49 of 2015 to week 19 of 2016) making it perhaps one of the longest running outbreaks of meningitis ever documented in recent times. It might indicate challenges with the surveillance system as observed when the outbreak first started in Tain measures were not taken to prevent the disease and allowing spread up to Jaman North (which finally reported the largest number of cases) indicating that the outbreak was always “a step ahead” of outbreak containment measures.

The six districts in which the cases were clustered are served by three of the largest hospitals in the Brong Ahafo region and these happen to be Christian Health Association of Ghana (CHAG) hospitals (have a history of high rates of utilization due to reputation built by early pioneering missionaries of these hospitals), Wenchi, Techiman, and Nkoranza. These hospitals serve a catchment area beyond the administrative districts in which they are located and thus had many cases reporting to them from neighbouring districts thus giving a picture of a belt which runs from Nkoranza south in the south east through Techiman, Wenchi and Tain to Jaman North in the north West part of the region.

The high mobility between the commercial towns of Techiman, Wenchi, and Nkoranza to the border town of Sampa might have also acted as a high risk/vulnerability factor in aiding the rapid spread of the disease. These districts lie at the fringes of the savannah ecological zone (which is known to have outbreaks of the disease) in the transitional zone of Ghana and with changing climatic conditions, is more prone to outbreaks of diseases hitherto restricted.

In our work the clinical management of cases and adherence to treatment protocols did not receive prominence thus we were unable to deduce the quality of care at each facility so as to draw a conclusion on case fatality rates. It might appear however that the hospitals which were located in the “Brong Ahafo meningitis belt” were better equipped to handle cases resulting in case fatalities lower than those (over 25%) in the 5 districts which share borders with the meningitis belt of Ghana [10]. The development of sore throat or symptoms of upper respiratory tract infection probably from the effects of the cold dry and dusty winds blowing during that period led to the development of sore thus creating easy portals of entry for organisms [4] and the eventual development of meningitis.

The association of meningitis and alcohol use could further be explored by our study if analysis could be in depth to in relation to other variables like occupation and onset of illness. This is because anecdotal evidence suggests a number of cases (with high fatalities) were linked to areas of illegal mining where there are also associated high levels of alcohol use [16]. A lumbar puncture rate of a little over 50% points to challenges in skills of clinicians, specimen collection and transportation, and above all the urge to treat before testing. It is not very whether the standard operating procedures for CSF collection and analysis during outbreaks were followed but gives rise to numbers and quality of lumbar punctures done at each facility to inform future training needs.

The challenge of the use of standard operating procedures during the outbreaks was noticed during the use of ceftriaxone in patient management without documentation of the efficacy and dosages used in the treatment of patients. The issues raised with efficacy might also be linked to the fact that Ceftriaxone alone is not adequate in the management of pneumococcal meningitis due to penicillin-resistant pneumococci [17].

The Ministry of Health had to [17, 18] send down another brand of ceftriaxone (after earlier brand were alleged to be ineffective against S. pneumoniae) [10] in order for clinicians and health facilities managers to gain confidence in antibiotics used during the outbreak.

It is also very difficult to document the effect of the prophylaxis with ciprofloxacin given out to contacts of cases as later events prove that it was not possible to conduct pharyngeal colonization surveys to determine the level of infectiousness of those individuals acting as carriers of the disease. The predominance of S. pneumoniae as the main causative organism in this outbreak confirms the work of other authors [4] as the organism gradually mimics or attains characteristics hitherto known to be exhibited by *N. meningitides* in its ability to cause epidemics. It also means that in the absence of any vaccination exercise which might have limited effect in such highly mobile populations a large number of carriers have developed and with possible harsh weather conditions in 2016/2017 are likely to see a repeat of this outbreak unless surveillance is enhanced.

Poor ventilation and the overcrowding of members of households sharing the same room during the nights are major contributory factors to the rapid spread of organisms causing meningitis as demonstrated by an average of 3 persons in each room.

A host of factors to include changes in climatic conditions, high mobility of carriers of the S. pneumoniae organism amongst and susceptible population and challenges with the surveillance system contributed to the outbreak of pneumococcal meningitis in the Brong Ahafo Region. Improved clinical capacity in the diagnosis and management of cases of meningitis, strengthening of the core and support functions of the integrated disease surveillance systems, and the improvement in community based documentation of health events are critical to either preventing or effectively managing an outbreak of pneumococcal meningitis in the Brong Ahafo Region in the near future.

## Conclusion

The study established a rare outbreak of meningitis due to S. pneumoniae in six districts of the Brong Ahafo region of Ghana. All age groups and sex were affected but mortality was higher in children less than 15 years. The outbreak peaked in epidemiological week 6 and lasted over 17 weeks. Upper respiratory infection prior to the outbreak and alcohol use were identified as factors that facilitated the transmission of pneumococcal meningitis in the region.

## Additional file

Additional file 1: Questionnaire. (DOC 104 kb)

## Abbreviations

BAR: Brong Ahafo Region
CHPS: Community Based Health Planning and Services
ERC: Ethical Review Committee
GFELTP: Ghana Field Epidemiology and Laboratory Training Programme
GHS: Ghana Health Service
OR: Odds Ratio
RHD: Regional Health Directorate
WHO: World Health Organization
