# Supplementary material for: Pneumococcal meningitis outbreak and associated factors in six districts of Brong Ahafo region, Ghana, 2016
Source: BMC Public Health. 2018 Jun 22;18:781. doi: 10.1186/s12889-018-5529-z (PMC6013862; doi:10.1186/s12889-018-5529-z)
Supplement: Supplementary file 1 — Questionnaire. (DOC 104 kb) [file 12889_2018_5529_MOESM1_ESM.doc]

**Ghana Health Service**

**Brong Ahafo Regional Health Directorate**

**Questionnaire on streptococcus isolation among residents of Tain district**

**Instruction: Please tick the correct answer in the box provided**

**ID #............................ Date …………………………**

| **Demographic data** | | | | | | |  |  |  |  |  |  |  |  |  |  |  |  |  |
| --- | --- | --- | --- | --- | --- | --- | --- | --- | --- | --- | --- | --- | --- | --- | --- | --- | --- | --- | --- |
| 1 | Age: ………. | | |  |  |  |  |  |  |  |  |  |  |  |  |  |  |  |  |
| 2 | Sex: | Male | |  | Female | | |  |  |  |  |  |  |  |  |  |  |  |  |
|  |  |  |  |  |  |  |  |  |  |  |  |  |  |  |  |  |  |  |  |
| 3 | Marital status: | | | |  |  |  |  |  |  |  |  |  |  |  |  |  |  |  |
|  | Single | |  | Divorced | | |  | Married | |  | Cohabitating | | | |  | others | |  |  |
| 4 | Occupation | | |  |  |  |  |  |  |  |  |  |  |  |  |  |  |  |  |
|  | Student | | |  | Farming | | |  | Trading | | |  | Mining | |  | Other | |  |  |
| 5 | Education | | |  |  |  |  |  |  |  |  |  |  |  |  |  |  |  |  |
|  | No formal education | | | | | |  | Primary | | |  | Secondary | | |  | Tertiary | |  |  |
| 6 | Religion | | |  |  |  |  |  |  |  |  |  |  |  |  |  |  |  |  |
|  | Christian | | |  | Muslim | |  | Traditional | | |  |  |  |  |  |  |  |  |  |
|  |  |  |  |  |  |  |  |  |  |  |  |  |  |  |  |  |  |  |  |
| **Suppressed immune system** | | | | | | | | | |  |  |  |  |  |  |  |  |  |  |
| 7 | Do you have any chronic medical condition? | | | | | | | | | | |  |  |  |  |  |  |  |  |
|  | Yes | |  | No |  |  |  |  |  |  |  |  |  |  |  |  |  |  |  |
| 8 | If yes, which of these? | | | | | |  |  |  |  |  |  |  |  |  |  |  |  |  |
|  | Diabetes | | | | Yes | |  | No |  |  |  |  |  |  |  |  |  |  |  |
|  | Alcoholism | | | | Yes | |  | No |  |  |  |  |  |  |  |  |  |  |  |
|  | HIV | | | | Yes | |  | No |  |  |  |  |  |  |  |  |  |  |  |
|  | Hypertension | | | | Yes | |  | No |  |  |  |  |  |  |  |  |  |  |  |
| 9 | Are you on any long term medication? | | | | | | | | |  |  |  |  |  |  |  |  |  |  |
|  | Yes | |  | No |  |  |  |  |  |  |  |  |  |  |  |  |  |  |  |
| 10 | If Yes which one? | | | | |  |  |  |  |  |  |  |  |  |  |  |  |  |  |
|  | Immunosuppressive | | | | | |  | Non-immunosuppressive | | | | | | |  |  | |  |  |
| 11 | Are you pregnant? | | | | |  |  |  |  |  |  |  |  |  |  |  |  |  |  |
|  | Yes | |  | No |  |  |  |  |  |  |  |  |  |  |  |  |  |  |  |
| 12 | have you been vaccinated against meningitis | | | | | | | | | | |  |  |  |  |  |  |  |  |
|  | Yes | |  | No |  |  |  |  |  |  |  |  |  |  |  |  |  |  |  |
| **Overcrowding** | | | | | | |  |  |  |  |  |  |  |  |  |  |  |  |  |
| 13 | How many people do you sleep with in the same room? | | | | | | | | | | | | | | ……………. | | |  |  |
|  |  |  |  |  |  |  |  |  |  |  |  |  |  |  |  |  |  |  |  |
| 14 | How many people are living in the household? | | | | | | | | | | | ………………………………… | | | | | |  |  |
|  |  |  |  |  |  |  |  |  |  |  |  |  |  |  |  |  |  |  |  |
| 15 | How many rooms are in the household? | | | | | | | | | | ………………………………………. | | | | | | |  |  |
|  |  |  |  |  |  |  |  |  |  |  |  |  |  |  |  |  |  |  |  |
| 16 | How many windows are in each room? | | | | | | | | | ……………………………………………… | | | | | | | |  |  |
|  |  |  |  |  |  |  |  |  |  |  |  |  |  |  |  |  |  |  |  |
| 17 | How many people do you work with at your workplace? | | | | | | | | | | | | | …………………. | | | |  |  |
|  |  |  |  |  |  |  |  |  |  |  |  |  |  |  |  |  |  |  |  |
|  |  |  |  |  |  |  |  |  |  |  |  |  |  |  |  |  |  |  |  |
| 18 | Have you attended any social gathering within the last two week? | | | | | | | | | | | | | | | |  |  |  |
|  | Yes | |  | No |  |  |  |  |  |  |  |  |  |  |  |  |  |  |  |
| 19 | what type of social gathering | | | | | | | |  |  |  |  |  |  |  |  |  | | |
|  | Religious | | |  | Marriage | | |  | Funeral | | |  | Community gathering | | | | | |  |
| 20 | Where did you attend the social gathering? | | | | | | | | | | |  |  |  |  |  |  |  |  |
|  | Within the township | | | | | |  |  |  | outside the township | | | | | |  |  |  |  |
| 21 | Have you travelled within the last two weeks? | | | | | | | | | | |  |  |  |  |  |  |  |  |
|  | Yes | |  | No |  |  |  |  |  |  |  |  |  |  |  |  |  |  |  |
|  |  |  |  |  |  |  |  |  |  |  |  |  |  |  |  |  |  |  |  |
| 22 | Where did you travelled to? ……………………………………… | | | | | | | | | | | | |  |  |  |  |  |  |

23. Smoking history; currently smokes, never smoked, previously smoked

24. Live with somebody who smokes. Yes, No

25. Cook in kitchen with firewood? Yes, No

26. Type of house made. Cement, Mud, other

27. Contact with known case in the past 2weeks? Yes, No

28 Had a running nose, flu, cold, sore throat two weeks prior?

29. Type of contact .Share a bedroom with a case, touched linings, eat together, touch deceased body, etc
